# Supplementary material for: End Sequence Analysis Toolkit (ESAT) expands the extractable information from single-cell RNA-seq data
Source: Genome Res. 2016 Oct;26(10):1397–410. doi: 10.1101/gr.207902.116 (PMC5052061; doi:10.1101/gr.207902.116)
Supplement: Supplemental Material [file supp_gr.207902.116_Supp_Materials.tar.gz › ESAT-esat_v0.1/doc/html/classbroad_1_1pda_1_1gene_1_1transcripts_null_model.html]

ESAT: broad.pda.gene.transcriptsNullModel Class Reference


ESAT


- Main Page
- Related Pages
- Packages
- Classes
- Files

- Class List
- Class Index
- Class Hierarchy
- Class Members

All Classes Namespaces Files Functions Variables Enumerator Pages

- broad
- pda
- gene
- transcriptsNullModel

Classes |
Public Member Functions |
List of all members

broad.pda.gene.transcriptsNullModel Class Reference

Collaboration diagram for broad.pda.gene.transcriptsNullModel:

[legend]

|  |  |
| --- | --- |
| Classes | |
| class | **chrSizeNode** |
|  | |

|  |  |
| --- | --- |
| Public Member Functions | |
|  | transcriptsNullModel (BEDFileParser refGenes, BEDFileParser centromers, String chrSizeF) throws IOException |
|  | |
| void | makeRandTranscriptList (int numRand, BEDFileParser ref) |
|  | |
| void | printRandModels (String outprefix) throws IOException |
|  | |
| void | writeIntergenicGenome (String outf) throws IOException |
|  | |
| ArrayList< BEDFileParser > | getRandomSets () |
|  | |
| void | CrossRandModelsWithBed (String bedToCrossWith\_fname, BEDFileParser refBed, String outfile) throws IOException |
|  | |

## Constructor & Destructor Documentation

|  |  |  |  |
| --- | --- | --- | --- |
| broad.pda.gene.transcriptsNullModel.transcriptsNullModel | ( | BEDFileParser | *refGenes*, |
|  |  | BEDFileParser | *centromers*, |
|  |  | String | *chrSizeF* |
|  | ) |  | throws IOException |

## Member Function Documentation

|  |  |  |  |
| --- | --- | --- | --- |
| void broad.pda.gene.transcriptsNullModel.CrossRandModelsWithBed | ( | String | *bedToCrossWith\_fname*, |
|  |  | BEDFileParser | *refBed*, |
|  |  | String | *outfile* |
|  | ) |  | throws IOException |

Here is the call graph for this function:

|  |  |  |  |  |
| --- | --- | --- | --- | --- |
| ArrayList<BEDFileParser> broad.pda.gene.transcriptsNullModel.getRandomSets | ( |  | ) |  |

Here is the caller graph for this function:

|  |  |  |  |
| --- | --- | --- | --- |
| void broad.pda.gene.transcriptsNullModel.makeRandTranscriptList | ( | int | *numRand*, |
|  |  | BEDFileParser | *ref* |
|  | ) |  |  |

Here is the call graph for this function:

|  |  |  |  |  |  |
| --- | --- | --- | --- | --- | --- |
| void broad.pda.gene.transcriptsNullModel.printRandModels | ( | String | *outprefix* | ) | throws IOException |

Here is the caller graph for this function:

|  |  |  |  |  |  |
| --- | --- | --- | --- | --- | --- |
| void broad.pda.gene.transcriptsNullModel.writeIntergenicGenome | ( | String | *outf* | ) | throws IOException |

---

The documentation for this class was generated from the following file:

- java/broad/pda/gene/transcriptsNullModel.java


---

Generated on Fri Aug 15 2014 15:11:00 for ESAT by  

 1.8.7
